# Translation reinitiation and development are compromised in similar ways by mutations in translation initiation factor eIF3h and the ribosomal protein RPL24

**DOI:** 10.1186/1471-2229-10-193

**Published:** 2010-08-27

**Authors:** Fujun Zhou, Bijoyita Roy, Albrecht G von Arnim

**Affiliations:** 1 Genome Science and Technology Program, The University of Tennessee, Knoxville, TN 37996, USA; 2 Department of Biochemistry, Cellular and Molecular Biology, The University of Tennessee, Knoxville, TN 37996, USA; 3 Laboratory of Gene Regulation and Development, Eunice Kennedy Shriver National Institute of Child Health and Human Development, Bethesda, MD 20892, USA

## Abstract

**Background:**

Within the scanning model of translation initiation, reinitiation is a non-canonical mechanism that operates on mRNAs harboring upstream open reading frames. The h subunit of eukaryotic initiation factor 3 (eIF3) boosts translation reinitiation on the uORF-containing mRNA coding for the Arabidopsis bZip transcription factor, AtbZip11, among others. The RPL24B protein of the large ribosomal subunit, which is encoded by *SHORT VALVE1*, likewise fosters translation of uORF-containing mRNAs, for example mRNAs for auxin response transcription factors (ARFs).

**Results:**

Here we tested the hypothesis that RPL24B and eIF3h affect translation reinitiation in a similar fashion. First, like *eif3h *mutants, *rpl24b *mutants under-translate the AtbZip11 mRNA, and the detailed spectrum of translational defects in *rpl24b *is remarkably similar to that of *eif3h*. Second, *eif3h *mutants display defects in auxin mediated organogenesis and gene expression, similar to *rpl24b*. Like AtbZip11, the uORF-containing ARF mRNAs are indeed undertranslated in *eif3h *mutant seedlings.

**Conclusion:**

We conclude that, similar to eIF3h, RPL24B bolsters the reinitiation competence of uORF-translating ribosomes. Coordination between eIF3 and the large ribosomal subunit helps to fine-tune translation of uORF-containing mRNAs and, in turn, to orchestrate plant development.

## Background

In eukaryotic cells, gene expression is highly regulated, often at multiple levels, such as transcription, mRNA structure and stability, translational control, and protein degradation. Translational regulation is arguably least well characterized, and questions concerning the mechanism of translational control abound. In metazoans and fungi, translation is regulated in response to nutritional and metabolic signals and in certain developmental contexts [1-3]. In plants, translation is regulated by small metabolites as well as environmental conditions [4-7].

According to the canonical model of eukaryotic translation, the ribosome dissociates from the mRNA for good as soon as it has terminated translation at a stop codon. However, there are exceptions to this rule. In Arabidopsis, about 30% of full-length mRNAs harbor one or more upstream open reading frames (uORFs) in their 5' leader sequence [8]. Once a uORF has been recognized and translated, the ribosome must resume scanning and reacquire its initiation factors in order to recognize the start codon of the main ORF, a process known as reinitiation. The efficiency of translational reinitiation is inversely related to uORF length. In yeast, for example, a 35-codon uORF all but abolishes translation downstream [9]. However, a small fraction of Arabidopsis uORFs exceeds this length. While uORFs generally inhibit translation, certain uORFs regulate translation in response to exogenous signals, for example, sucrose or polyamines [5,10,11].

In Arabidopsis, carboxyl-terminal truncation alleles of *eIF3h *cause under-translation of specific mRNAs, many of which harbor multiple upstream open reading frames (uORFs) in their 5' leader [8,12]. The h subunit of eukaryotic translation initiation factor, eIF3, ameliorates the inhibitory effect of specific uORFs in part by promoting the reinitiation competence of the translating ribosome [13].

Among the eukaryotic translation initiation factors, eIF3 is by far the most complex. It consists of 12 subunits in Arabidopsis [14]. The functions of the individual eIF3 subunits remain to be fully characterized. eIF3 participates in almost all major steps during initiation, such as tRNA charging of the 40S ribosomal subunit; and loading of the charged 40S onto the mRNA's 5' Untranslated Region (UTR) [15]. eIF3 also affects mRNA scanning and start codon recognition [16-18]. Moreover, eIF3 may help with dissociating post-termination ribosomes into their large and small subunits and thereby facilitate ribosome recycling [19]. The h subunit of eIF3 is not conserved in budding yeast, but forms part of the functional core of mammalian eIF3 [20].

Aside from eIF3h, another plant-encoded protein that fosters translation of uORF-containing mRNAs is the large ribosomal protein, RPL24B. Deletion mutations in *RPL24B/SHORT VALVE (STV*) cause defects in organ initiation, vascular patterning, and gynoecium structure, which have been attributed in part to undertranslation of mRNAs for transcription factors of the auxin response factor (ARF) class [21]. Auxin plays critical roles in the initiation and specification of postembryonic organs emerging from the apical meristems as well as in the establishment of the apical-basal axis [22-24]. The short-range directional auxin transport governs primordium initiation on the shoot apical meristem (SAM), thereby affecting phyllotaxis [25,26]. While PIN proteins guide directional auxin transport, ARFs are transcriptional regulators that convert the local auxin concentration into a gene expression response (reviewed by [27]). Among the latter, ARF5/MONOPTEROS helps to establish the apical-basal axis [28,29], whereas ARF3/ETTIN has multiple roles in defining the dorsoventrality of leaves and in gynoecium development [30-32].

Here we address the question whether RPL24B and eIF3h contribute to reinitiation in distinct ways or similar ways. As a model system, we utilize the 5' leader of the Arabidopsis *bZip11 *gene, which harbors 4 uORFs. Of these, the second is strongly inhibitory to translation and is physiologically important because it mediates a translational repression by sucrose [7,10,11,13]. We demonstrate that mutations in *RPL24B *and *eIF3h *have a similar spectrum of defects in the translation of a panel of mutant versions of the AtbZip11 mRNA. In addition, like *rpl24b/stv1*, *eif3h *mutant plants display defects in auxin responses. And, finally, *eif3h *mutant plants undertranslate the uORF-containing mRNAs for several ARF auxin response factors, including *ARF3 *and *ARF5*, which are clients of RPL24B. These data raise the strong possibility that the initiation factor eIF3 cooperates with the large ribosomal subunit in bringing about translation reinitiation.

## Results

### A mutant of *RPL24B *displays similar translation defects as does *eif3h*

**Figure 1:**
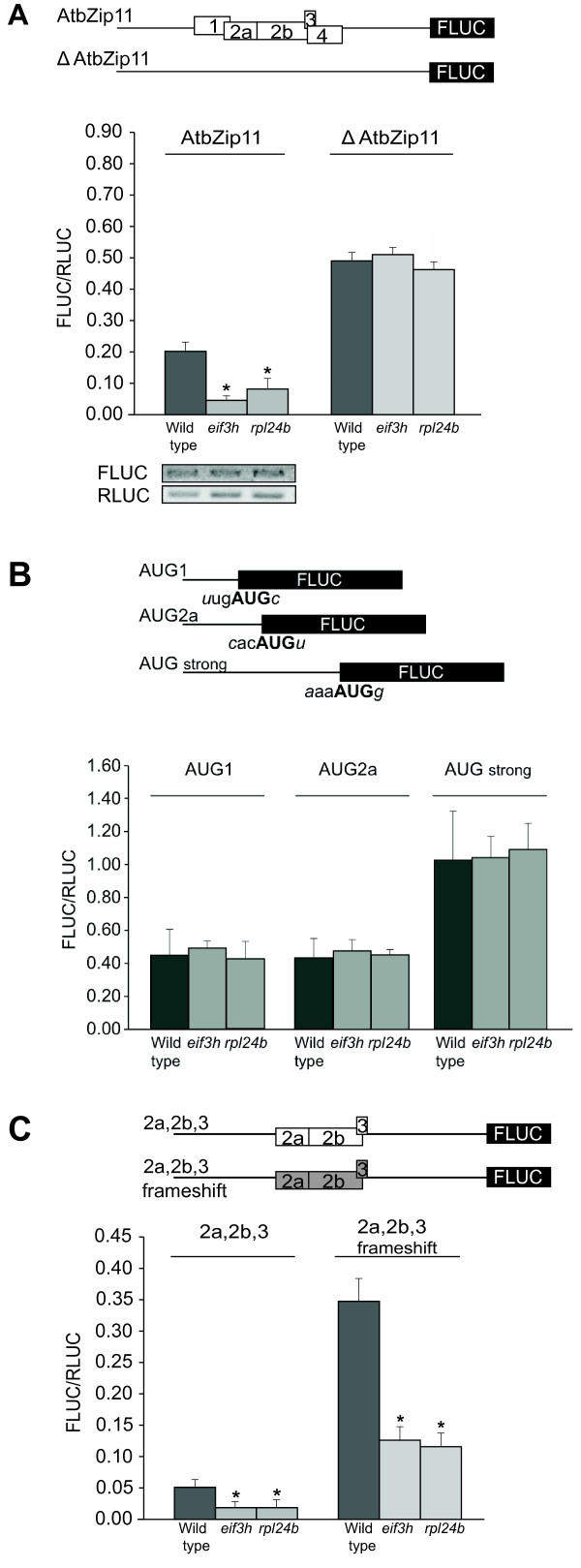
**Both eIF3h and RPL24B are required for efficient translation of the uORF-containing *AtbZip11 *mRNA**. Data are from transient dual-luciferase gene expression assays in ten day old seedlings. The respective 5' leader is fused to firefly luciferase and is expressed in the presence of a reference gene expressing *Renilla *luciferase as an internal control for transformation efficiency. Both genes are transcribed from a CaMV 35S promoter [13]. **(A) **Schematic of the 5' leader of the *AtbZip11 *mRNA. Boxes numbered 1 to 4 represent uORFs. In the *Δ AtbZip11 *mutant the five uAUGs are replaced with stop codons [13]. Bars denote standard error; n = 7 to 10; * P < 0.002 by Student's t-test when compared to wild type. The gel images below the graph show mRNA levels for FLUC and RLUC as determined by subsaturating RT-PCR. Gel lanes correspond to the bars in the graph above. **(B) **Recognition of the uAUG start codons in *AtbZip11*. Like *eif3h *[13], *rpl24b *is not defective in recognizing the weak start codons, uAUG1 and uAUG2a, nor a strong version of uAUG4. **(C) **Testing the dependence of translation on the uORF peptide sequence. Both *rpl24b *and *eif3h *are inhibited to a similar degree by uORFs encoding the original uORF2 and uORF3 peptide sequences (white box in the schematic) and alternative peptide sequences generated by site-directed mutagenesis and a pair of compensatory frame shift mutations (gray box). For details on plasmid construction see [13].

**Figure 2:**
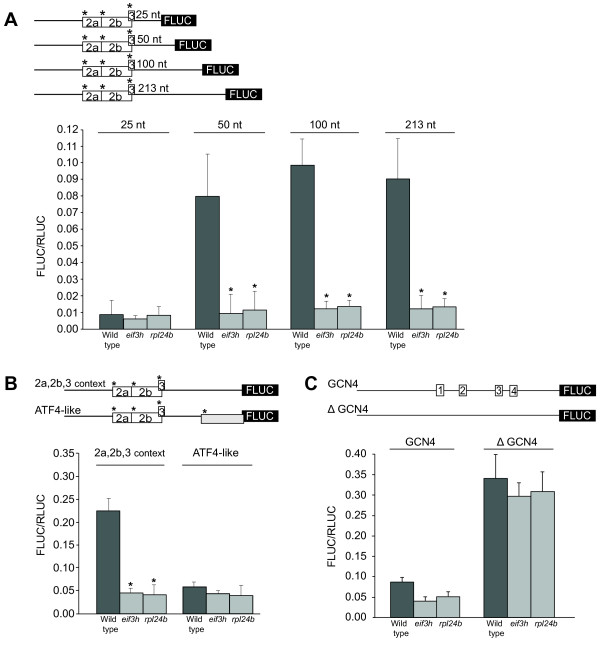
**RPL24B is required alongside eIF3h for efficient reinitiation after uORF translation**. **(A) **Schematic of the uORF pattern in four constructs that differ in the length of the intercistronic spacer [13]. uORFs 2a 2b and 3 each commence with a AUG codon in a strong Kozak context (asterisks) to minimize leaky scanning. Translational efficiency was estimated as previously described for Fig. 1. The spacer length dependence of reinitiation in the wild type is thought to reflect that acquisition of a fresh methionyl-tRNA by the 40S ribosome requires a minimal scanning time [54]. Notably, *rpl24b *exhibits a similar reinitiation defect as *eif3h*. Bars denote standard error; n = 5-10; * P < 0.002 by Student's t-test. **(B) **The ATF4-like leader contains an additional uAUG in a strong context (A_-3_AAAUGG_+4_) that leads into an overlap-uORF. The new AUG lies 150nt downstream of the uORF 2/3 cluster and 60nt upstream of the FLUC AUG. Both *rpl24b *and *eif3h *mutants show poor FLUC expression in the presence or absence of the overlap uORF. Bars represent standard error (n = 8-12). **(C) **The similarity in translational activity between *rpl24b *and *eif3h *can also be seen on the 5' leader from yeast GCN4 when expressed heterologously in Arabidopsis (n = 5; bars denote standard error).

The *stv1-1 *deletion mutation truncates the *RPL24B *gene, and is henceforth referred to as *rpl24b*. We examined whether *rpl24b *displays gene expression defects similar to the carboxyl-terminal deletion allele of eIF3h, *eif3h-1*. Indeed, translational efficiency on the uORF-containing mRNA of Arabidopsis bZip11 was reduced in *rpl24b*, similar to *eif3h-1 *(Fig. 1A). Notably, the reporter gene was expressed normally when the uORFs were removed. In previous research, we had employed a series of two dozen mutated versions of the AtbZip11 5' leader in order to narrow down the likely molecular defect in *eif3h *[13]. This series of constructs was used here to compare *rpl24b *with *eif3h*. In the first set of constructs, individual uAUGs in the context of the AtbZip11 leader were fused directly to the FLUC coding sequence in order to compare uAUG recognition. *Rpl24b *mutants did not display any defect in start codon recognition, whether the AUG was in a weak or strong context (Fig. 1B). Next, we addressed whether reinitiation after uORF2 and 3 was sensitive to the uORF peptide sequence. The uORF2 peptide sequence is inhibitory to translation in wild type and *eif3h *[13]. The coding sequence of uORF2 and 3 was changed using compensatory frameshift mutations and additional site-directed mutagenesis [13]. The data show that *rpl24b *mutants were as sensitive as *eif3h *mutants to the coding sequences of the uORF2 and uORF3 peptides (Fig. 1C). In the following, we tested the dependence of reinitiation on the length of the spacer between uORFs and main ORF. While wild-type plants were able to reinitiate translation downstream of a simplified uORF cluster as long as the spacer sequence was equal to or longer than 50 nucleotides, *rpl24b *mutants could not reinitiate here, again similar to *eif3h *mutant plants (Fig. 2A). One might hypothesize that the *rpl24b *or *eif3h *mutations cause a relaxation of start codon recognition specifically for reinitiating ribosomes. If so, the defect in the mutants would be due - not to a failure to resume scanning but - to premature initiation at non-AUG codons in the 213nt spacer sequence, which could easily cause a failure to recognize the AUG start codon of FLUC. In this case, shortening the spacer sequence would have ameliorated the mutant defect, resulting in increased FLUC expression. Evidently, this was not the case. It stands to reason that neither the *eif3h *mutant nor *rpl24b *have relaxed start codon recognition.

The constructs shown in Fig. 2B demonstrated the negative effect on translation when a uORF overlaps the main ORF, a situation found in the mRNA for the mammalian bZip protein, ATF4 [33]. The reduction of translation by the overlapping uORF in the wild type points to the fact that wild-type ribosomes reinitiate after translating the upstream uORF. In contrast, in *eif3h*, and evidently also in *rpl24b*, translation was poor even in the absence of the ribosome-trapping overlapping uORF, suggesting that reinitiation after the first uORF is poor. The yeast GCN4 5' leader is another example of a 5' leader that requires reinitiation for full expression [34], although the GCN4 uORFs are short and translational attenuation in *eif3h *is less pronounced [13]. Again, *rpl24b *behaved similarly to *eif3h *(Fig. 2C). Taken together, all these data suggest that RPL24B and eIF3h have closely related molecular functions during translation reinitiation.

### Many *eif3h *mutant phenotypes are reminiscent of defects in auxin transport or response

**Figure 3:**
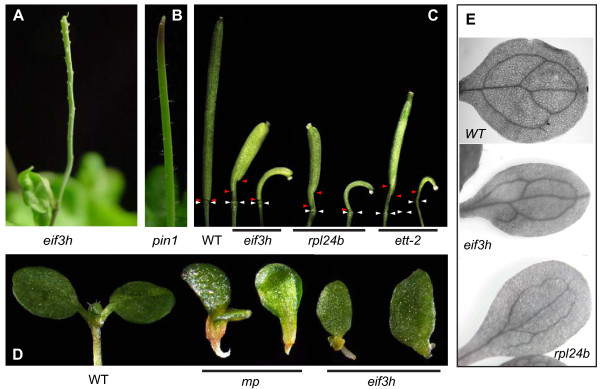
***eif3h *phenotypes reminiscent of defects in auxin transport or response**. **(A) ***eif3h *naked shoot. Compared to the canonical pins of the *pin1 *mutant **(B)**, *eif3h *pins tended to bear scaly, rudimentary organs. **(C) ***eif3h *siliques show defects in carpel development, similar to those of *rpl24b/stv1-1 *or *ett-2*, an allele of *ARF3*. White arrowheads point to the last node carrying stamens and red arrowheads point to the basal end of the carpel valves. **(D) **Rare monocotyledon phenotype of *eif3h *seedlings, similar to that of *mp*, an allele of *ARF5*. **(E) **Cotyledons were cleared to reveal defects in vascular development. Similar defects as in *eif3h *can be seen in *rpl24b/stv1-1 *[21].

The emerging hypothesis that RPL24B cooperates with eIF3h predicts that mutations in the two genes have overlapping developmental phenotypes. Instead of a functional inflorescence with leaves and flowers, *eif3h *mutant plants sometimes formed a pin-formed shoot, especially when shifted from short-day to long-day growth conditions (20%, n = 90) (Fig. 3A). Pin-formed shoots are characteristic of defects in auxin transport or response, for example the *pin1 *mutant (Fig. 3B[27]) and mutations in the auxin response transcription factor, MONOPTEROS (MP)/ARF5 [35]. These data suggest that eIF3h may boost translation of proteins needed for auxin transport or auxin response. In further support of auxin response defects, some *eif3h *mutant seedlings had only a single cotyledon or two cotyledons of unequal size, similar to the *mp *(*arf5*) mutant (Fig. 3D) and *rpl24b/stv1 *[21]. Auxin defects often reveal themselves by cul-de-sac vascular elements in the cotyledons, and such defects were readily observed in the *eif3h *mutant (Fig. 3E).

The siliques of the *eif3h *mutant were shorter than wild type, and typically the valves initiated at an unusual distance from the node. Occasionally a valve was missing from one side of the silique, which is reminiscent of the *ettin/arf3 *mutant (Fig. 3C; [30,31]). In keeping with the hypothesis that eIF3h cooperates with RPL24B, *rpl24b/stv1 *displayed similar valve defects ([21] Fig. 3C). Notably, the *rpl24b *mutants will also display pin-formed shoots when enhanced by an *ettin/arf3 *mutation [21].

**Figure 4:**
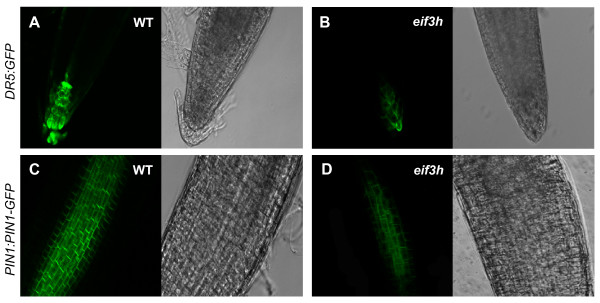
**Auxin-responsive reporter gene expression in *eif3h-1 *mutants**. **(A **and **B) ***DR5:GFP *expression in wild type and *eif3h *root tips, respectively. **(C **and **D) ***PIN1:PIN1-GFP *expression in wild type and *eif3h *mutant root, respectively. Left panels show GFP fluorescence by confocal microscopy of a medial optical section and right panels show the corresponding brightfield image. Paired images were taken under the same magnification, exposure time, and processing conditions.

To address the postulated auxin response defects in the *eif3h *mutant at a cellular level *in situ*, the auxin responsive *DR5:GFP *gene and a *PIN1-GFP *gene under the control of the *PIN1 *promoter (*PIN1:PIN1-GFP*) were crossed into the *eif3h *mutant. While *DR5:GFP *was highly expressed in the wild-type root tip, it was significantly reduced in the *eif3h *mutant (Fig. 4A, B). Likewise, the *PIN1:PIN1-GFP *expression was significantly reduced in the *eif3h *mutant roots (Fig. 4C, D). The potential for low expression of the *PIN1 *auxin efflux carrier in the *eif3h *mutant presents a possible explanation for the appearance of pin-formed inflorescence shoots.

### eIF3h boosts translation of uORF-containing *ARF *mRNAs

**Figure 5:**
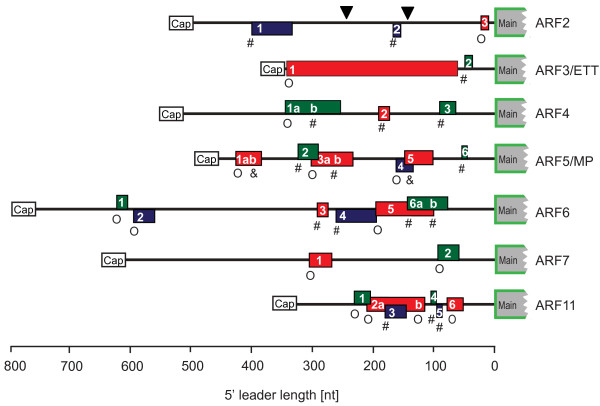
**Long 5' leaders of many auxin response factor (ARF) mRNAs harbor multiple uORFs**. Rectangles represent uORFs that are in frame (red) with the main ORF, in the -1 position (green), or in the +1 position (blue). Start codon contexts are illustrated as weak (O, NNNAUGN), moderate (#, RNNAUGN or NNNAUGG) and strong (&, RNNAUGG). The cDNA sequences shown generally correspond to the longest known gene model displayed at http://www.arabidopsis.org/  in November 2009: *ARF2 *(AT5G62000.1); *ARF3 *(AT2G33860.1); *ARF4 *(AT5G60450.1); *ARF5 *(AT1G19850.1); *ARF6 *(AT1G30330.1); *ARF7 *(AT5G20730.1); *ARF11 *(AT2G46530.1). A splice variant known for *ARF2 *retains uORF1 and uORF3 (intron flanked by black triangles).

eIF3h counteracts the translational repression by uORFs [8,12,13]. Among auxin-related genes, most *ARF*s harbor multiple uORFs. *ARF5*, *ARF6*, and *ARF11 *each have seven or more upstream AUGs (uAUGs) (Fig. 5). In contrast, uORFs were uncommon among *AUX/IAA *and *YUCCA *mRNAs, *TIR1 *auxin receptor homologs, and *PIN *mRNAs (Table 1). The abundance of uORFs in the *ARF *5' leaders indicates that these mRNAs are potential clients of eIF3h.

**Table 1:** uAUG abundance in 5' leader sequences of auxin related mRNAs

Gene family	Leader sequences available	No. of uAUG/leader	No. of uAUG/100nt	% Leaders with 2 or more uAUG	% Leaders with 3 or more uAUG
ARF	14	3.3	0.9	64	36
YUCCA	3	1.0	0.3	33	0
AFB/TIR1-like	5	0.8	0.3	20	0
AUX/IAA	26	0.4	0.3	12	0
PIN	6	0.16	0.16	0	0

Note: Data based on the longest mRNA 5' leader sequence available in the Genome Browser at the *Arabidopsis *Information Resource http://www.arabidopsis.org/ on June 27, 2009.

**Figure 6:**
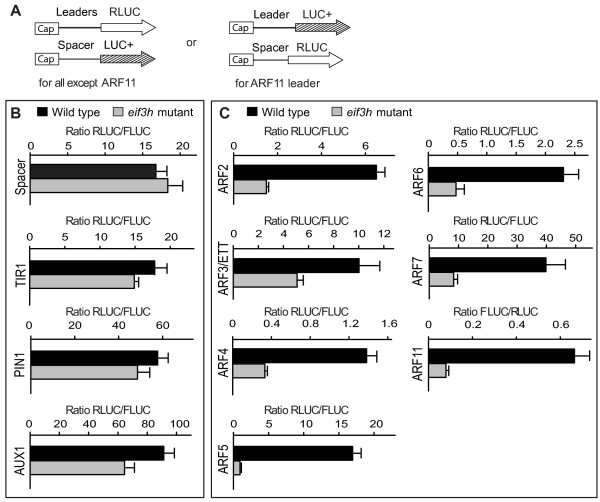
**The 5' leaders of many uORF-containing *ARF *mRNAs render translation dependent on eIF3h**. **(A) **Schematic view of the mRNAs for protoplast transformation. Open arrows represent RLUC ORF, hatched arrows represent codon-optimized FLUC (LUC+). mRNAs were prepared by *in vitro *transcription with SP6 RNA polymerase. An equal amount of internal control (Spacer-LUC+) mRNA was added to the 5' leader-RLUC mRNA to be tested as an internal control for transformation efficiency. **(B and C) **Translational efficiency on the given ARF 5' leader is expressed as the mean RLUC/FLUC ratio with standard errors from three replicate transformations. Data from several other auxin related 5' leaders are shown for comparison. *PIN1 *does not contain uORFs. *AUX1 *and *TIR1 *each have one short uORF of six codons.

A protoplast transformation assay based on *in vitro *transcribed mRNA was adopted to observe the translation efficiency of specific mRNA 5' leaders in the *eif3h *mutant. While translation with a *PIN1 *leader, which lacks uORFs, or *TIR1 *and *AUX1 *leaders with only one short uORF, was not dramatically affected, the *ARF *leaders with multiple uORFs were poorly translated in the *eif3h *mutant (Fig. 6B, C). Translation of ARF3 and ARF5 were previously shown to be dependent on RPL24B. Results shown here now indicate that both eIF3h and RPL24B are required for maximal translation of the same target mRNA.

## Discussion

### Coordination between 60S subunit and eIF3 during reinitiation

Reinitiation can be defined as translation initiation downstream of a uORF by a ribosome that has just terminated translation at the uORF termination codon. It is the small (40S) subunit that reinitiates, while the 60S subunit is most likely recycled. The efficiency of reinitiation varies depending on the sequence context in and around the uORF. Reinitiation can be quite efficient when the uORF is short. Reinitiation becomes less efficient as the length of the uORF increases or as the time necessary to translate it increases. Translation of a regular protein-coding ORF will typically abolish the reinitiation competence [3,36]. The mechanism of reinitiation is not well understood. Compared to regular, cap-dependent, initiation, one predicts the following unique aspects as the small subunit of the ribosome resumes scanning. First, the 40S subunit of the ribosome resumes scanning without apparent contact to the cap binding protein eIF4E. Second, the 40S is not charged with a tRNA or ternary complex and must reaquire it. And third, the 40S lacks several other initiation factors, such as eIF1, eIF1A, both of which should have been ejected from the 40S during 40S-60S subunit joining. eIF1 and 1A may, however, rejoin the 40S subunit after termination to participate in 40S-60S subunit separation [19].

Many case studies of translation reinitiation have implicated eIF3 in the process. eIF3h [8] and eIF3a [18] help when uORFs are short or generic. Other studies [12,13,37] attribute a role to eIF3 for reinitiation after longer uORFs. Reinitiation after long ORFs is rare and requires either specialized RNA sequence elements [38] or specialized reinitiation factors [39-41]. Yet, we suspect that eIF3 is required in these cases as well. Specifically, the reinitiation motif of feline calicivirus RNA binds to eIF3 [38]. And the reinitiation factor, TAV, of cauliflower mosaic virus interacts with the g subunit of eIF3 [39], aside from a newly discovered plant protein termed re-initiation supporting protein (RISP) [41]. These and other data have given rise to the hypothesis that eIF3 remains attached to the 40S for a few codon-cycles of translation elongation [e.g. [3,13,18,37]], although direct biochemical evidence for this notion is still lacking.

Meanwhile, circumstantial evidence indicates that reinitiation also requires proteins of the large ribosomal subunit [42]. The uORFs in the ARF3 and ARF5 mRNAs are less inhibitory when RPL24B is intact [21]. Second, the CaMV reinitiation factor, TAV, interacts not only with eIF3g but also with RPL24 [39], although it has not yet been shown that mutations of eIF3g or RPL24 will impede the reinitiation activity of TAV.

The experiments presented here further test the hypothesis that a subunit of the 60S ribosome, RPL24B, and a subunit of eIF3 cooperate to foster reinitiation. We newly demonstrate that mutations in both *rpl24b *and *eif3h *inhibit reinitiation of the same uORF-containing mRNA, AtbZip11. Moreover, the ARF3 and ARF5 mRNAs, which are poorly translated in *rpl24b*, are also poorly translated in *eif3h*, alongside with other uORF-studded ARF mRNAs. These findings would predict that *rpl24b *and *eif3h *share visible developmental phenotypes, which is indeed the case. Both display defects in the development of the cotyledon vasculature and the valves of the fruit, a phenotype characteristic of *arf3/ettin *mutants. The pin-formed inflorescences that can form in *eif3h *mutants, which are characteristic of certain *arf5 *alleles [35], were not seen in *rpl24b *per se, but were seen when *rpl24b *was enhanced by an *arf3 *mutation [21]. Although the phenotype of *rpl24b *is generally less dramatic than that of *eif3h*, all together, these findings provide new evidence for a close functional interaction between eIF3 and the 60S subunit.

The mode of interaction between eIF3h and RPL24 could be physically direct or indirect, or they may act independently. Concerning a direct interaction, we consider that RPL24B is located at the leading edge (the side of mRNA entry) of the 60S subunit, and more specifically at the 40S/60S interface where it forms the intersubunit bridge B7 toward the long helix 44 in the 40S subunit [43]. Meanwhile, eIF3 binds to the lagging edge of the 40S subunit just below the mRNA exit channel [44]. Thus, assuming that eIF3 might remain bound to an 80S ribosome, eIF3h and RPL24 would reside on opposite faces of the ribosome and would be far apart. Thus, a direct interaction seems unlikely because it would require that eIF3 detach from the 40S, or, possibly, that the interaction take the form of a bridge between separate 60S and 40S subunits that are not part of the same 80S ribosome. An indirect interaction between RPL24 and eIF3h would occur if eIF3 was attached to an 80S ribosome. Under this circumstance, it may seem surprising that mutations in two remote components of this molecular machine would affect reinitiation in similar ways. However, it is worth to point to related precedents. For example, mutations in several, physically separate, RPL proteins affect plant development in remarkably similar ways [45]. Finally, eIF3h and RPL24 might bolster reinitiation independently. For example, eIF3h might bolster the competence to resume scanning after uORF termination [13], and RPL24 might affect elongation. Slowing elongation on a uORF can reduce the efficiency of reinitiation [36].

### Translational control of development

The developmental defects identified in the *eif3h *mutants underscore that translational control by uORFs might play an important role in modulating auxin responses during plant development. Defects in the carpel valves in *rpl24b/stv1 *were interpreted as a consequence of the undertranslation of ARFs [21], and this phenotype likewise appeared in *eif3h *mutants. Mutants with defects in auxin action typically have defects in vascular development, as seen here in *eif3h*. Because *ARF*s activate transcription from the *DR5 *promoter [46], one expects underexpression of *DR5:GFP*, which was in fact observed in the *eif3h *mutant (Fig. 4A, B), indicating a reduced auxin response. Moreover, because *ARF5/MP *stimulates *PIN1 *expression and thus auxin efflux [46,47], the undertranslation of *ARF5 *in the *eif3h *mutant (Fig. 6) would cause underexpression of *PIN1:PIN1-GFP*, as well as a tendency for pin-formed shoots, as was indeed observed (Fig. 4C, D; Fig. 3A). Upregulation of several *AUX/IAA *mRNAs in the *eif3h *mutant [12] may also contribute to reduce the *PIN1:PIN1-GFP *expression [48]. Taken together, the auxin related growth defects of the *eif3h *mutant may be caused in part by reduced translation of *ARF*s. Mutations affecting ribosomal proteins other than RPL24 also compromise development [45,49]; identifying whether the underlying mechanisms operate in conjunction with eIF3 will be a topic for future investigation.

## Conclusions

This study indicated that both eIF3h and RPL24 promote the translation of uORF-containing mRNAs. The *rpl24b *and *eif3h-1 *mutants displayed similar translational defects on the *AtbZip11 *mRNA and its variants, as well as on mRNAs for auxin response transcription factors. The two mutants also showed similar auxin-related developmental defects. The translation initiation factor eIF3h may play an important role in translational control by communicating with RPL proteins and thus enhancing the reinitiation competence of the large subunit of the ribosome.

## Methods

### Plant growth conditions

Growth conditions for wild type (Wassiliweskija ecotype) and the *eif3h*-1 allele (At1g10840), which harbors a T-DNA insertion in the 10th of 12 exons, have been described [12]. The *rpl24b *mutant allele (At3g53020) is allele *stv1-1 *(Arabidopsis Biological Resource Center stock number CS6957) in the Wassiliweskija ecotype. *DR5:GFP *and *PIN1:PIN1-GFP *transgenes were introduced into the *eif3h-1 *background and Wassiliweskija wild-type by crossing.

### Molecular cloning

The plasmids for in vitro transcription were made in the TA-cloning vector pKRX [50] and contained the SP6 phage promoter, the translational leader from tobacco etch virus (TL) and the coding region of firefly luciferase (FLUC) or LUC+ (from pGL3-basic, Promega, Madison, WI) or Renilla luciferase (RLUC; [51]) followed by a 70 nucleotide long poly-A tail. The TL 5' leader was replaced with the respective leader sequence to be assayed. The 5' leader called Spacer is the multiple cloning site of pGL3-basic.

### Microscopy

GFP fluorescence was visualized on a Leica SPI 2 laser scanning confocal microscope.

### DNA based expression assay after transient transformation

Wild-type, *rpl24b/stv1-1 *and *eif3h-1 *mutant plants were grown for ten days on MS agar plates with 1% sucrose. Plasmids carrying dual-luciferase constructs were introduced by particle bombardment as previously described [12]. Transformed seedlings were incubated at 22°C in a lighted growth chamber for 8 hours before assaying for luciferase activity. Activities of the experimental luciferase and the reference luciferase were measured in a single protein extract using the Dual Luciferase system (Promega, Madison, WI) in the TD-20/20 luminometer (Turnerdesigns, Sunnyvale, CA). Mean ratios of experimental and reference luciferase from at least 3 biological replicates were used to compare the translation efficiency between wild type and *eif3h *mutant.

### Protoplast preparation and mRNA transformation

Protoplasts were prepared from shoots of wild-type or mutant 7-day-old Arabidopsis seedlings [52] and were transformed with 200ng mRNA using the polyethyleneglycol method [53] as described [13]. The protoplasts were incubated in a 24 well plate for 3 hours in the dark at room temperature, then harvested by centrifugation for luciferase assays.

## Authors' contributions

FZ performed developmental and gene expression studies for Figs. 3, 4, 5 and 6 and Table 1, BR carried out gene expression assays in Fig. 1-2. AGV directed the research and wrote the manuscript. All authors read and approved the final manuscript.
